# Efficacy and Safety of Cryobiopsy vs. Forceps Biopsy for Interstitial Lung Diseases, Lung Tumors, and Peripheral Pulmonary Lesions: An Updated Systematic Review and Meta-Analysis

**DOI:** 10.3389/fmed.2022.840702

**Published:** 2022-03-10

**Authors:** Mohan Giri, Guichuan Huang, Anju Puri, Rongjuan Zhuang, Yishi Li, Shuliang Guo

**Affiliations:** ^1^Department of Pulmonary and Critical Care Medicine, The First Affiliated Hospital of Chongqing Medical University, Chongqing, China; ^2^Department of Pulmonary and Critical Care Medicine, The First People's Hospital of Zunyi (The Third Affiliated Hospital of Zunyi Medical University), Zunyi, China; ^3^Department of Nursing, The First Affiliated Hospital of Chongqing Medical University, Chongqing, China

**Keywords:** cryobiopsy, forceps biopsy, interstitial lung diseases, lung tumors, meta-analysis, peripheral pulmonary lesions, transbronchial cryobiopsy

## Abstract

**Background:**

Cryobiopsy has emerged as a novel alternative to conventional forceps biopsy for the diagnosis of interstitial lung diseases (ILDs), lung tumors, and peripheral pulmonary lesions (PPLs). This study aims to compare cryobiopsy and forceps biopsy for the diagnosis of these lung pathologies with respect to efficacy and safety by performing a meta-analysis of updated evidence.

**Methods:**

A number of databases, such as PubMed, Embase, Web of Science, the Cochrane Library, OVID, CNKI, and Wanfang database, were searched for eligible studies. Randomized and non-randomized comparative studies investigating the efficacy and safety of cryobiopsy vs. forceps biopsy for lung pathologies were included. Pooled results were calculated as an odds ratio (*OR*) or standardized mean difference (SMD) with 95% *CI*.

**Results:**

A total of 39 studies, such as 9 RCTs with 3,586 biopsies (1,759 cryobiopsies and 1,827 flexible forceps biopsies) were analyzed. Cryobiopsy was associated with a significant increase in the diagnostic rates of ILDs (*OR*, 4.29; 95% *CI*, 1.85–9.93; *p* < 0.01), lung tumors (*OR*, 3.58; 95% *CI*, 2.60–4.93; *p* < 0.01), and PPLs (*OR*, 1.70; 95% *CI*, 1.23–2.34; *p* < 0.01). Cryobiopsy yielded significantly larger specimens compared with flexible forceps biopsy (SMD, 3.06; 95% *CI*, 2.37–3.74; *p* < 0.01). The cryobiopsy group had a significantly higher (moderate to severe) bleeding risk than the forceps group (*OR*, 2.17; 95% *CI*, 1.48–3.19; *p* < 0.01). No significant difference was observed in the incidence of pneumothorax between the groups (*OR*, 0.90; 95% *CI*, 0.44–1.85; *p* = 0.78).

**Conclusion:**

Our results demonstrate that cryobiopsy is a safe and efficacious alternative to conventional forceps biopsy.

## Introduction

Conventional transbronchial forceps biopsy (TBFB) is still a universal tissue sampling technique for histopathological analysis of various lung pathologies. Transbronchial forceps biopsy has been used in conjugation with brushing, needle biopsy, or washings to increase diagnostic sensitivity (1). Unfortunately, this combined technique deemed to increase overall cost and procedural time. Furthermore, the diagnostic utility of specimens obtained by forceps is limited by inadequate sample size and crush artifacts, which affect the quality of the histological analysis. Surgical lung biopsy (SLB), the current gold standard diagnosis approach, has been very successful in obtaining significantly larger samples with fewer artifacts, thus permitting a histologic diagnosis in more than 90% of cases (2). The 2018 revised clinical practice guidelines by the American Thoracic Society, European Respiratory Society, Japanese Respiratory Society, and Latin American Thoracic Society made a conditional recommendation for surgical lung biopsy in the diagnosis of interstitial lung diseases (ILDs) (3). However, concerns remain regarding the morbidity and mortality, as the in-hospital mortality for SLB ranges from 1.7% for elective procedures to 16% for non-elective procedures (2).

To overcome these limitations, cryotechnology has been introduced as an important tool in the therapeutic armamentarium for endobronchial tumors, cryoextraction of malignant airway stenosis, mechanical tumor debulking, and cryosurgery for lung carcinoma (4–7). Subsequently, cryotherapy technique led to the development of cryoadhesion and cryorecanalization. In recent years, cryobiopsy has entered the bronchoscopic arena due to its ability to obtain larger biopsies without crush artifact, more alveolar tissue, and better diagnostic yield compared with conventional biopsy techniques. Cryobiopsy currently is an emerging modality in the diagnostic armory for ILD, peripheral pulmonary lesion (PPL), endobronchial masses, and surveillance for acute cellular rejection after lung transplantation (8–11). Evidence on the efficacy and safety of cryobiopsy is evolving, but transbronchial cryobiopsy (TBCB) procedures seem to be associated with increased bleeding compared with the forceps biopsies (12). We previously published a meta-analysis on the efficacy and safety of cryobiopsy vs. forceps biopsy for ILD and lung tumors that showed cryobiopsy as a superior diagnostic tool with larger specimen area than forceps biopsy (13). After 5 years of our published article, we decided to update our meta-analysis based on the new clinical evidence available and further verify the efficacy and safety of cryobiopsy for the diagnosis of ILDs, lung tumor, and PPLs.

## Methods

### Search Strategy

This systemic review and meta-analysis was conducted in accordance with the preferred reporting items for systematic reviews and meta-analysis (PRISMA) statement. We searched seven databases (PubMed, Embase, Web of Science, the Cochrane Library, OVID, CNKI, and Wanfang database) for studies published until October 18, 2021. We searched combinations of the terms [(Cryobiopsy OR Cryoprobe biopsy OR Cryotransbronchial biopsy OR Transbronchial cryobiopsy) AND (lung cancer OR lung neoplasm OR lung carcinoma OR lung tumor OR lung adenocarcinoma) OR (pulmonary nodule OR peripheral pulmonary lesion) OR (interstitial lung diseases OR diffuse lung diseases OR lung fibrosis)]. Additionally, we reviewed the reference lists of retrieved articles for any relevant studies.

### Eligibility Criteria and Selection Process

**Figure 1:**
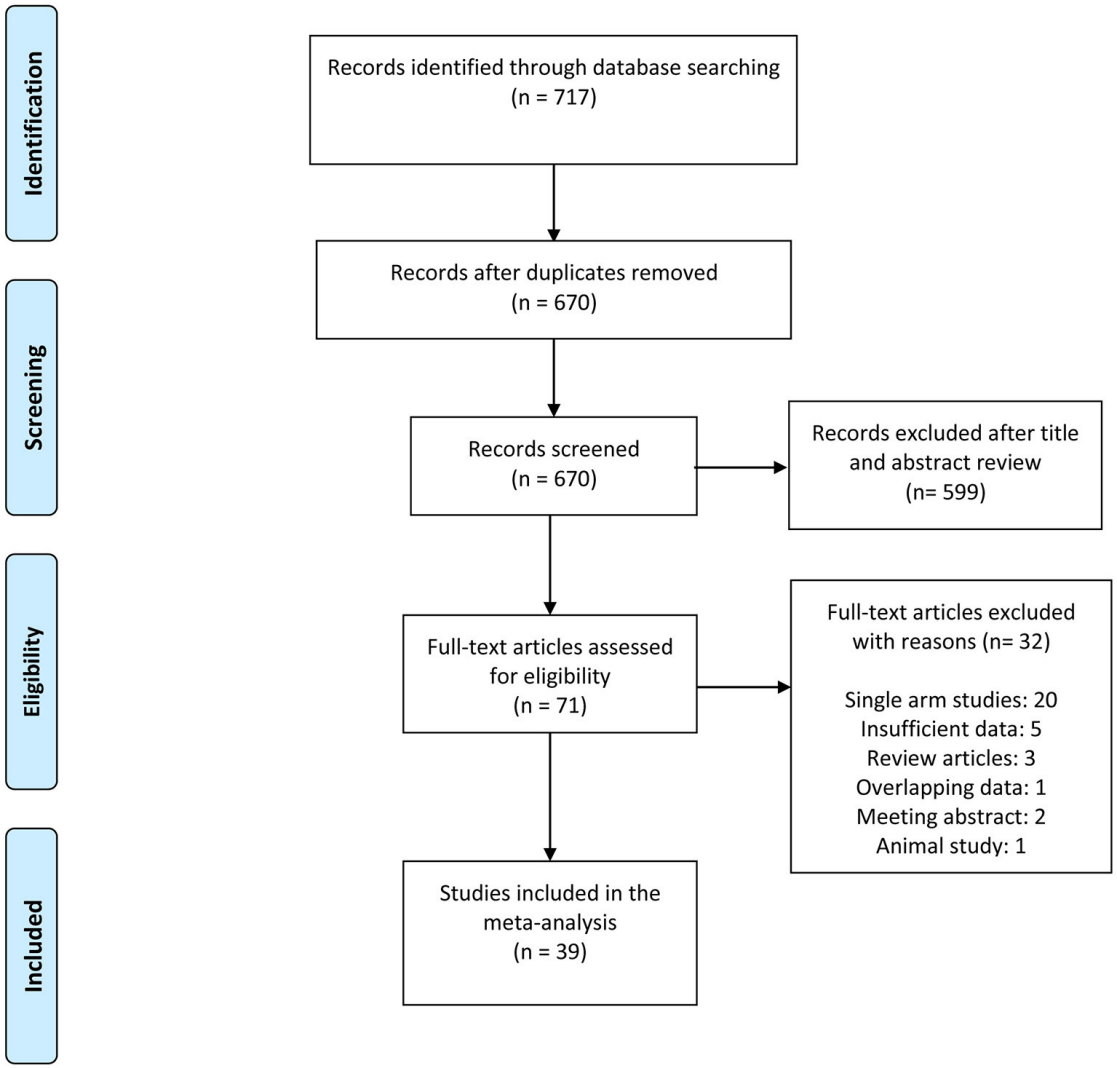
Preferred reporting items for systematic reviews and meta-analysis (PRISMA) flowchart of the study selection process.

The inclusion criteria were as follows: (1) randomized controlled trials (RCTs) or cohort studies (retrospective or prospective studies) with ten or more subjects; (2) articles that compared cryobiopsy vs. forceps biopsy for the diagnosis of ILDs, lung tumors, and PPLs. We excluded review articles, non-comparative studies, letters, and conference papers. Non-English studies were also considered if they met the inclusion criteria. Two independent reviewers (MG and GCH) screened the title and abstract according to inclusion and exclusion criteria. Discrepancies in study inclusion were resolved by consulting a third reviewer. Kappa statistic was used to measure an inter-rater agreement between two primary reviewers. The selection process is summarized in the flowchart in Figure 1.

### Data Extraction and Quality Assessment

We collected data in a standardized way by using a predesigned spreadsheet. The following data were retrieved from all included studies: first author, year of publication, country of data collection, study type, specimen size, diagnostic rate, bleeding severity, and complications. Discrepancies during data extraction were resolved by consensus. The original authors of studies were contacted if relevant data were not available in the published reports. The methodological quality of non-randomized controlled trials (NRCT) were assessed with the Newcastle-Ottawa Scale (NOS) by two authors (MG and GCH) (14). The maximum score on the NOS is 9. NOS scores of 0–3, 4–6, and 7–9 were considered to indicate low, moderate, and high quality, respectively. Cochrane Collaboration's tool was used to assess the potential sources of bias in RCTs (15). The Seven sources of bias were assessed: random sequence generation, allocation concealment, blinding of participants and personnel, blinding of outcome assessment, incomplete outcome data, selective reporting, and other bias. For each individual domain, studies were classified as having low, unclear, or high-risk of bias. In case of disagreement, a consensus was reached through discussion with a third reviewer. Data provided as median and interquartile range (IQR) were converted to calculate the mean and SD according to Wan et al. (16).

### Outcome Measures

The efficacy and safety endpoints were analyzed. The efficacy endpoints were diagnostic yield, specimen size; the safety endpoint were bleeding severity and incidence of pneumothorax.

### Statistical Analysis

All analyses were performed in “meta” package of R software Version 4.1.0 (https://www.r-project.org/) and Review Manager, version 5.3 (Cochrane Collaboration). Dichotomous variables were analyzed for odds ratios (*OR*s) with 95% *CI*s, and continuous variables were presented as standardized mean difference (SMD) with 95% *CI*s. The magnitude of statistical heterogeneity between studies was quantified by using *I*^2^ statistics, *I*^2^ values of 25, 50, and 75% were considered low, moderate, and high heterogeneity, respectively. We constructed funnel plots to assess publication bias. Additionally, we assessed publication bias *via* Begg's and Egger's tests (the *p* less than 0.05 indicates publication bias) and the trim and fill method. The values of *p* < 0.05 were considered statistically significant.

## Results

### Description of Included Studies

The literature search yielded 717 studies, of which 71 were selected for further evaluation using the full text. Of the remaining 71 articles, 32 were excluded after full-text review. A total of 39 studies were included in the final analysis. The detailed literature search process is presented in Figure 1. A total of 39 studies, such as 9 RCTs (11, 12, 17–23), 16 retrospective studies (4, 9, 24–37), 13 prospective studies (10, 38–49), and 1 cohort study (50) representing 3,586 biopsies (1,759 cryobiopsies and 1,827 flexible forceps biopsies) were analyzed. The mean age of the study patients ranged from 45 to 69.5 years. Furthermore, 16 of the studies were conducted in the China, whereas 23 studies were conducted outside China. The summary of included studies is presented in Table 1. Diagnostic rate was reported for 36 studies, while 25 studies included data on specimen size. It was shown that 23 studies provided data on the bleeding severity and 9 studies provided data on the incidence of pneumothorax.

**Table 1:** Characteristics of included studies.

**References**	**Design**	**Country**	**Disease type**	**Age, mean ±**	**Men/**	**Cryobiopsy yield**	**Forceps biopsy**	**NOS**
				**SD or median**	**Women**	**(diagnostic/**	**(diagnostic/**	**score**
				**(range) years**		**total cases)**	**total cases)**	
Aktas et al. (10)	Prospective	Turkey	Lung tumor	57.83 ± 10.88	37/4	38/41	32/41	7
Arimura et al. (45)	Prospective	Japan	PPL	69.5 (46–82)	20/3	20/23	19/23	8
Babiak et al. (9)	Retrospective	USA	ILD	NR	NR	39/41	24/41	6
Chen and Zhan (30)	Retrospective	China	Lung tumor	58 ± ?	52/13	54/65	44/65	6
Chen et al. (44)	Prospective	China	ILD	51 ± 13	16/9	20/25	3/25	6
Chou et al. (4)	Retrospective	China	Lung tumor	64 (49–76)	48/27	75/75	52/75	6
Cirak et al. (37)	Retrospective	Turkey	ILD	58.37 ± 9.33	44/38	45/82	75/82	6
Ding et al. (48)	Prospective	China	ILD	NR	12/8	7/20	1/20	6
Ehab et al. (21)	RCT	Egypt	Lung tumor	55.47 ± 11.57	32/15	35/47	24/47	–
El-Assal et al. (20)	RCT	Egypt	Lung tumor	60.25 ± 6.48	40/0	20/20	17/20	–
El-Dahdouh et al. (46)	Prospective	Egypt	Lung tumor	57.04 ± 6.4	18/7	25/25	20/25	8
Griff et al. (43)	Prospective	Germany	PPL	NR	NR	NR	NR	6
He et al. (29)	Retrospective	China	PPL	64 ± 11.2	40/36	33/37	27/39	7
Hetzel et al. (19)	RCT	Germany	Lung tumor	NR	424/169	268/282	239/281	–
Hetzel et al. (12)	RCT	Germany	ILD	62.8 ± 14	198/153	NR	NR	–
Hibare et al. (36)	Retrospective	India	PPL	NR	37/18	19/28	21/28	6
Huang et al. (24)	Retrospective	China	Lung tumor	63 ± ?	38/11	46/49	37/49	6
Huang et al. (17)	RCT	China	PPL	NR	25/15	11/20	12/20	–
Imabayashi et al. (35)	Retrospective	Japan	PPL	66.9 ± 10.3	15/20	31/36	24/29	6
Jiang et al. (34)	Retrospective	China	PPL	NR	38/21	21/28	20/31	7
Jiang (25)	Retrospective	China	Lung tumor	58.2 ± 6.7	28/24	45/52	33/52	6
Kho et al. (33)	Retrospective	Malasiya	PPL	58.5 (49.8–68.3)	78/36	18/24	20/41	7
Kim et al. (42)	Prospective	South Korea	Lung tumor	62.1 ± 9	25/5	27/30	23/30	6
Koslow et al. (50)	Cohort	USA	ILD	61 ± 14	143/128	66/120	62/151	8
Liu et al. (32)	Retrospective	China	ILD	45 ± 16	21/33	44/54	23/54	6
Li et al. (26)	Retrospective	China	ILD	49.6 ± 14.9	22/14	15/17	13/36	6
Lv et al. (18)	RCT	China	PPL	NR	69/63	44/65	38/65	–
Nasu et al. (31)	Retrospective	Japan	PPL	75 (41–90)	34/19	45/53	46/53	6
Pajares et al. (11)	RCT	Spain	ILD	NR	36/41	29/39	13/38	–
Pajares et al. (41)	Prospective	Spain	ILD	65.7 ± 11.9	72/52	59/124	24/124	8
Pang et al. (40)	Prospective	China	Lung tumor	62.5 (41.7–78.3)	26/14	37/40	28/40	7
Schumann et al. (22)	RCT	Germany	Lung tumor	63.4 ± 11.8	225/71	49/55	36/55	–
Schumann et al. (23)	RCT	Germany	PPL	68 ± ?	28/11	23/31	19/31	–
Shafiek et al. (39)	Prospective	Egypt	ILD	NR	8/17	10/12	5/13	7
Tao et al. (49)	Prospective	China	ILD	69.22 ± 7.84	32/28	14/30	5/30	7
Taton et al. (38)	Prospective	Belgium	PPL	68 ± 9	18/14	20/29	11/29	7
Torky et al. (47)	Prospective	Spain	PPL	NR	NR	NR	NR	6
Xiang et al. (28)	Retrospective	China	ILD	NR	16/13	10/14	7/15	6
Zhou et al. (27)	Retrospective	China	PPL	NR	33/22	21/26	16/29	6

*ILD, interstitial lung disease; NR, not reported; NA, not applicable; NOS, Newcastle-Ottawa Scale; PPL, peripheral pulmonary lesion; RCT, randomized controlled trial*.

*The meaning of the symbol “?” is value is not reported or provided*.

### Quality of the Studies

The NOS scores were ≥6 for all the NRCTs. The average NOS score of the included studies was 6.53 (range 6–8). All NRCTs were deemed to be moderate to high methodological quality with a low-risk of bias (Supplementary Table 1). The risk of bias graph for included RCTs using the Cochrane risk of bias tool is summarized in Supplementary Figure 1 and the risk of bias for each included study is included in Supplementary Figure 2. The overall quality of the included RTCs was found to be adequate. Seven of the 9 studies included had an unclear or high risk of selection bias. Eight of the 9 studies had a high or unclear risk of performance bias. Three studies had a high risk of detection bias and one study had a high risk of reporting bias. Cohen's kappa value of 0.823 indicated that an inter-rater agreement for the quality assessment of included studies between both reviewers was fair.

### Efficacy Endpoints

#### Diagnostic Rate

**Figure 2:**
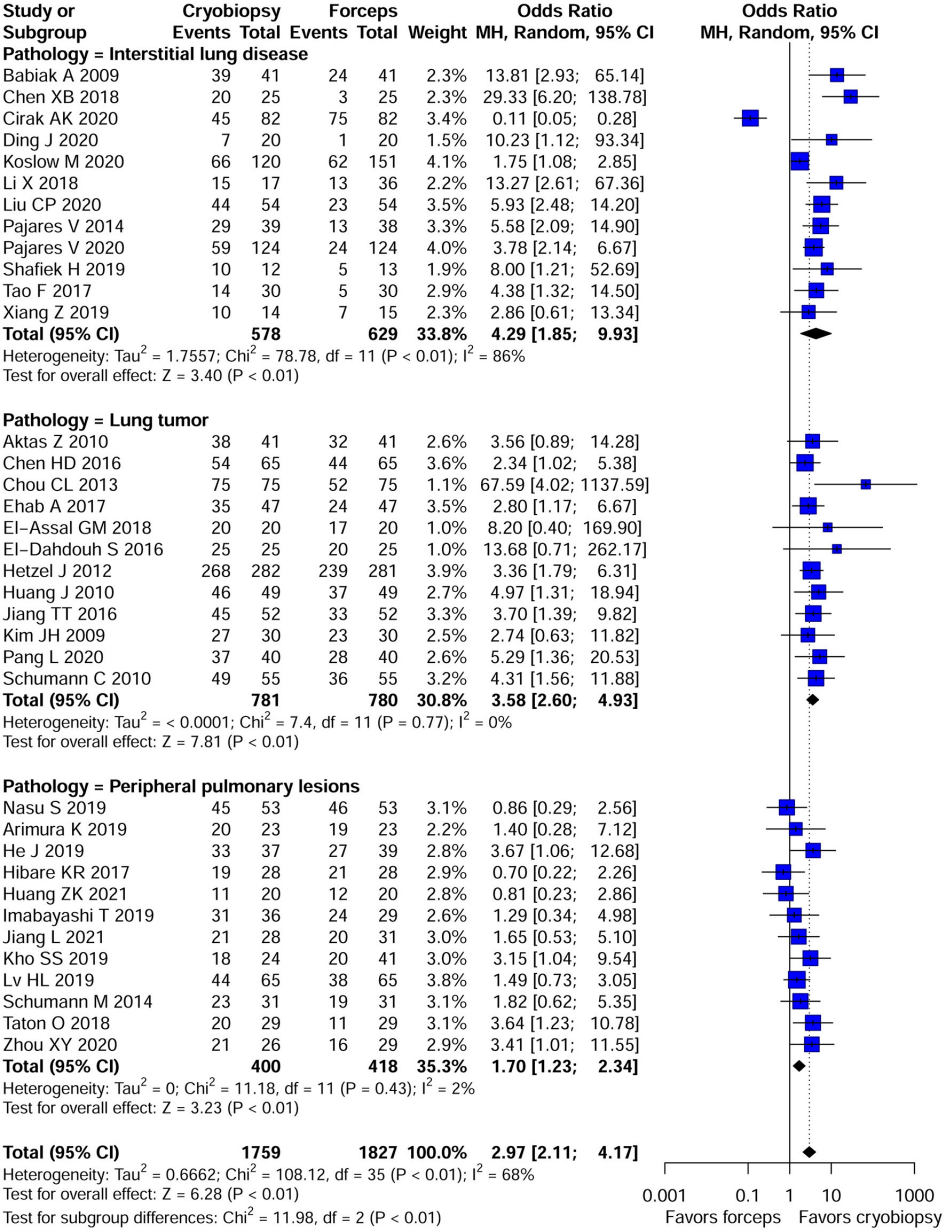
Forest plot of diagnostic yield of cryobiopsy vs. forceps biopsy. Subgroup analysis showed that compared with forceps biopsy, cryobiopsy was associated with a significant increase in the diagnostic rates of interstitial lung diseases (ILDs) (*p* < 0.01), lung tumors (*p* < 0.01), and peripheral pulmonary lesions (PPLs) (*p* < 0.01). CI, confidence interval.

The overall pooled *OR* was 2.97 (95% *CI*, 2.11–4.17; *p* < 0.01; *I*^2^ = 68%) with the diagnostic yield of 78.6% for cryobiopsies and 60.9% for flexible forceps, when including data across all studies. There was significant difference between the two techniques in terms of diagnostic rate (Figure 2). We included three lung pathologies (ILDs, lung tumors, and PPLs) in this meta-analysis. On subgroup analysis stratified by pathologies, cryobiopsy was associated with a significant increase in the diagnostic rates of ILDs (*OR*, 4.29; 95% *CI*, 1.85–9.93; *p* < 0.01; *I*^2^ = 86%), lung tumors (*OR*, 3.58; 95% *CI*, 2.60–4.93; *p* < 0.01; *I*^2^ = 0%), and PPLs (*OR*, 1.70; 95% *CI*, 1.23–2.34; *p* < 0.01; *I*^2^ = 2%) (Figure 2).

A subgroup analysis was also performed to test the diagnostic yield of cryobiopsies and flexible forceps stratified by study design (RCTs vs. NRCTs), study location (Chinese vs. non-Chinese studies), and year of publication (studies published between 2008 and 2018 vs. studies published between 2019 and 2021). When analyzing data from RCTs and NRCTs, the pooled *OR*s were 2.60 (95% *CI*, 1.71–3.94; *p* < 0.01; *I*^2^ = 33%) and 3.17 (95% *CI*, 2.06–4.86; *p* < 0.01; *I*^2^ = 72%), respectively. Subgroup analyses by study location for diagnostic rate showed significantly higher diagnostic rate in the Chinese studies compared with the non-Chinese ones (*OR*, 3.82; 95% *CI*, 2.50–5.84 vs. *OR*, 2.37; 95% *CI*, 1.46–3.84; *p* < 0.01). Subgroup analyses are presented in Table 2. We examined the publication bias of all 36 studies that compared the diagnostic yield of cryobiopsy and forceps biopsy using the funnel plot. The visual inspection of funnel plot revealed asymmetry (Supplementary Figure 3). Egger's (*p* = 0.0649) test also showed that there was evidence of publication bias. A sensitivity analysis was performed using the trim and fill method, the funnel plot became symmetrical after imputing the ten unpublished studies (Supplementary Figure 3). The overall pooled OR remained statistically significant at 2.13 (95% *CI*, 1.50–3.02; *p* < 0.001) (Supplementary Figure 4). Correction for potential publication bias had no significant effect on the pooled estimate.

**Table 2:** Summarized meta-analysis results of subgroup analysis.

**Variable**	**Number of**	**Cryobiopsy**	**Forceps**	**OR/SMD**	**95% CI**	**Heterogenity**	**I^2^^§^**	**Meta-analysis**
	**studies**		**biopsy**			***P* value**		***P* value**
**Subgroup analysis by diagnostic rate**								
Lung pathologies
ILD	12	61.9%	40.5%	4.29	(1.85, 9.93)	<0.01	86	**<0.01**
Lung tumors	12	92.1%	75%	3.58	(2.60, 4.93)	0.77	0	**<0.01**
PPLs	12	76.5%	65.3%	1.70	(1.23, 2.34)	0.43	2	**<0.01**
Study design
RCTs	8	85.7%	71.5%	2.60	(1.71, 3.94)	0.16	33	**<0.01**
Non-RCTs	28	75.3%	56.3%	3.17	(2.06, 4.86)	<0.01	72	**<0.01**
Study location
Chinese studies	16	80.6%	55.7%	3.82	(2.50, 5.84)	0.01	50	**<0.01**
Non-Chinese studies	20	77.6%	63.8%	2.37	(1.46, 3.84)	<0.01	74	**<0.01**
Year of publication
2008–2018	19	87.8%	66.7%	3.96	(2.83, 5.53)	0.04	39	**<0.01**
2019–2021	17	67.1%	53.9%	2.03	(1.21, 3.40)	<0.01	76	**<0.01**
**Subgroup analysis by specimen size**								
ILD	8	-	-	2.86	(1.89, 3.83)	<0.01	90	**<0.01**
Lung tumors	6	-	-	2.97	(1.84, 4.09)	<0.01	93	**<0.01**
PPLs	8	-	-	3.33	(1.84, 4.82)	<0.01	96	**<0.01**
**Subgroup analysis by moderate to severe bleeding**								
ILD	8	-	-	2.47	(1.10, 5.56)	<0.01	62	**<0.05**
Lung tumors	7	-	-	1.92	(1.22, 3.04)	0.15	36	**<0.01**
PPLs	4	-	-	2.58	(0.59, 11.35)	0.10	52	0.21
**Subgroup analysis by incidence of pneumothorax**								
ILD	5	4.7%	3.5%	1.60	(0.52, 4.93)	0.20	34	0.42
PPLs	4	9.4%	15.2%	0.55	(0.26, 1.19)	0.33	12	0.13

*OR, odds ratio; 95%CI. 95% confidence interval; ILDs, interstitial lung disease; PPLs, peripheral pulmonary lesions; RCTs, randomized controlled trials, SMD, standardized mean difference*.

§ *I^2^ index to quantify the degree of heterogeneity*.

*The meaning of the bold value are statistically significant*.

#### Specimen Size

**Figure 3:**
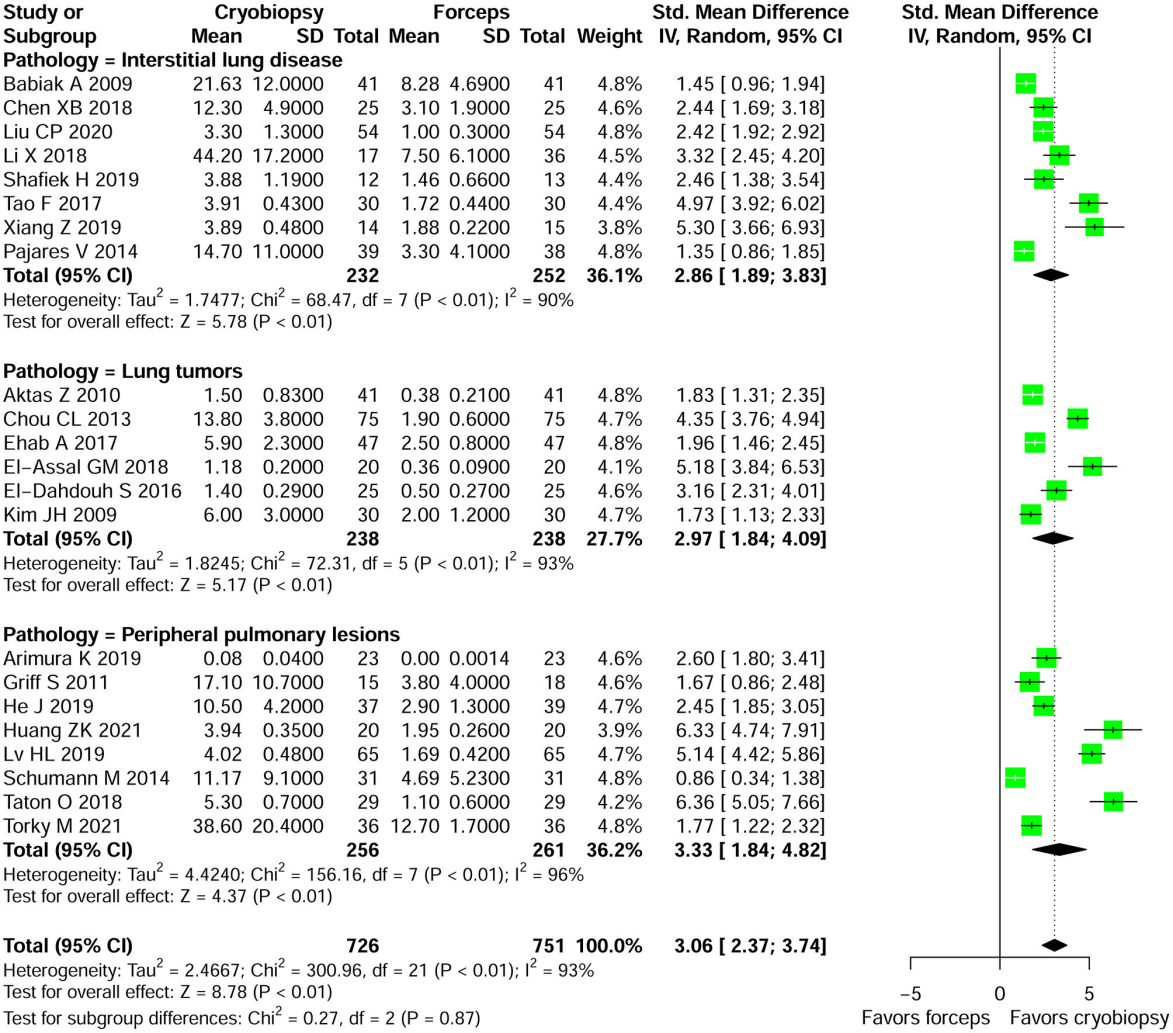
Forest plot of specimen size obtained by cryobiopsy and forceps biopsy. Subgroup analysis stratified by lung pathologies (such as, ILDs, lung tumors, and PPLs) showed that there were significant differences in specimen size between the cryobiopsy and forceps biopsy in all three subgroups (*p* < 0.01). CI, confidence interval; SD, standard deviation; SMD, standardized mean difference.

Supplementary Table 3 summarizes the results of specimen size obtained by cryobiopsy and forceps biopsy. A qualitative analysis showed that specimen size was larger in cryobiopsy samples as compared with forceps biopsy. Among the studies that provide data on specimen size, we were only able to perform meta-analysis of 22 studies. Cryobiopsy yielded significantly larger specimens compared with flexible forceps biopsy (pooled SMD: 3.06; 95% *CI*, 2.37–3.74; *p* < 0.01; *I*^2^ = 93%) (Figure 3). However, there was a significant heterogeneity in the studies that reported this outcome. A subgroup analysis stratified by lung pathologies, such as ILDs, lung tumors, and PPLs showed that there were significant differences in specimen size between the cryobiopsy and forceps biopsy in all three subgroups (*p* < 0.01) (Figure 3). Based on the visual inspection of the funnel plot (Supplementary Figure 5) and quantitative measurement that used the Egger's test (*p* < 0.001) and Begg's test (*p* < 0.01), there was significant publication bias. We used the trim and fill method (Supplementary Figure 5) to account for the influence of publication bias, and the result of SMD (1.93, 95% *CI*: 1.05–2.82, *p* < 0.01) (Supplementary Figure 6) confirmed that publication bias had no significant impact on the pooled estimate.

### Safety Endpoints

#### Bleeding Severity

**Figure 4:**
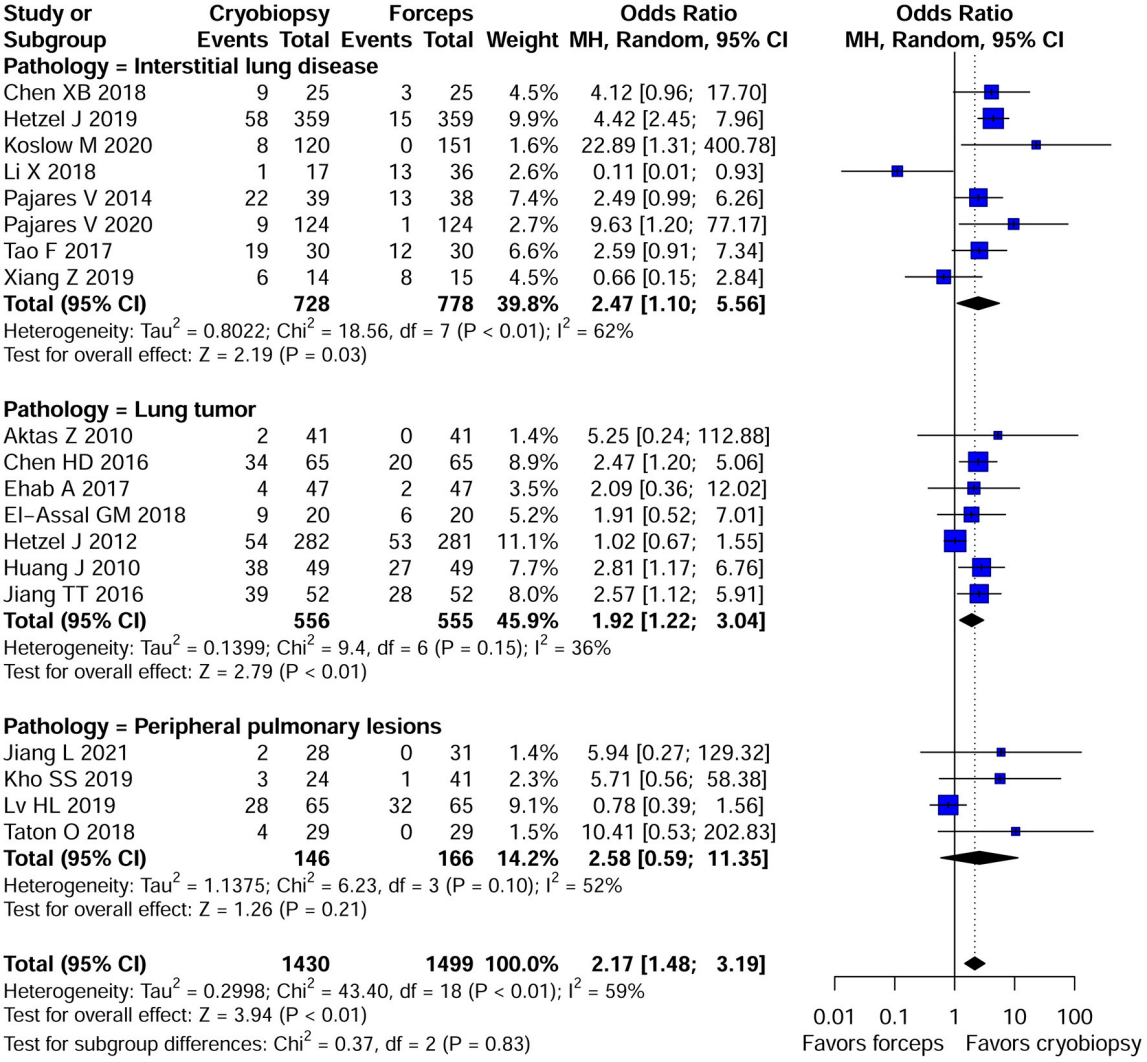
Forest plot of moderate to severe bleeding between the cryobiopsy and forceps biopsy. Subgroup analysis revealed that there were significant differences in the moderate to severe bleeding between the cryobiopsy and forceps biopsy in ILDs and lung tumors subgroups (<0.05) but not in PPLs subgroup (*p* > 0.05). CI, confidence interval.

There was substantial variation among the studies regarding the definition of bleeding severity. Qualitative analyses of bleeding severity in included studies is summarized in Supplementary Table 2. We performed the meta-analysis of 19 studies that provided data regarding moderate to severe bleeding and had similar definitions of bleeding severity. The risk for moderate to severe bleeding was higher in patients who underwent cryobiopsy than those who underwent forceps biopsy (*OR*, 2.17; 95% *CI*, 1.48–3.19; *p* < 0.01; *I*^2^ = 59%) (Table 2; Figure 4). In addition, a subgroup analysis showed that there were significant differences in the moderate to severe bleeding between the cryobiopsy and forceps biopsy in ILDs (*OR*, 2.47; 95% *CI*, 1.10–5.57; *p* = 0.03; *I*^2^ = 62%) and lung tumors (*OR*, 1.92; 95% *CI*, 1.22–3.04; *p* < 0.01; *I*^2^ = 36%) subgroups but not in PPLs (*OR*, 2.58; 95% *CI*, 0.59–11.35; *p* = 0.21; *I*^2^ = 52%) subgroup (Figure 4). Symmetrical funnel plot (Supplementary Figure 7) and the quantitative assessments by Egger's test (*p* = 0.32) and Begg's test (*p* = 0.34) revealed no evidence of publication bias.

#### Incidence of Pneumothorax

Only 9 studies reported the incidence of pneumothorax with cryobiopsy and forceps biopsy. The incidence of pneumothorax was not statistically different between the cryobiopsy and forceps biopsy groups (*OR*, 0.90; 95% *CI*, 0.44–1.85; *p* = 0.78; *I*^2^ = 34%) (Supplementary Figure 8). Subgroup analysis showed no significant difference in the incidence of pneumothorax between cryobiopsy and forceps biopsy in both the ILDs (*p* = 0.42) and PPLs (*p* = 0.13) subgroups (Table 2; Supplementary Figure 8). The Egger's (*p* = 0.36) and Begg's (*p* = 0.67) tests found no evidence of publication bias, and the funnel plot showed a roughly symmetrical distribution of studies (Supplementary Figure 9).

## Discussion

Biopsy specimen is often required during the diagnostic processes of ILDs, lung tumors, and PPLs, as the radiological and clinical findings are not sufficient to allow for confident clinical diagnosis. A study has demonstrated that, up to 30% of the patients with ILDs necessitate histopathological evaluation of the lung to establish the diagnosis (51). TBCB has emerged as a novel tool that often helps to overcome many limitations of currently used sampling methods, such as transthoracic fine needle aspiration, surgical lung biopsy, and TBFB. Cryobiopsy enables the acquisition of larger specimen with more alveolated tissue and reduced tissue artifacts. However, concerns have been raised over the increased risk of bleeding and pneumothorax during cryobiopsy procedure. Recent chest guideline and expert panel report (52) recommended that TBCB may be considered as an alternative to surgical lung biopsy in patients with suspected ILD, by adopting a precautionary measure, such as prophylactic use of a bronchial blocker and fluoroscopy. Data are limited on the usefulness of cryobiopsy compared with forceps biopsy for lung pathologies. Given the widespread use of TBCB for the etiological diagnosis of lung pathologies, there is a clear need to systematically evaluate the impact of this novel sampling technique in clinical practice.

To the best of our knowledge, our study is the most comprehensive meta-analysis to date evaluating cryobiopsy vs. forceps biopsy for lung pathologies. The results of our meta-analysis are robust as we included 39 studies with more than three times the number of patients of previous meta-analysis (13). We showed that the diagnostic yield was significantly higher in cryobiopsy than forceps biopsy group irrespective of lung pathologies. However, there is significant heterogeneity across studies that is likely driven by variations in study design, sample size, and procedural aspects. Although the surgical lung biopsy (SLB) has long been considered the gold standard for the diagnosis of diffuse parenchymal lung disease, in recent years, lung cryobiopsy has been adopted into clinical practice for the diagnosis of lung pathologies. A systematic review and meta-analysis including 11 studies showed diagnostic yield for the transbronchial lung cryobiopsy of approximately 80% in patients with suspected ILD (53). Although there was significant heterogeneity across studies, another meta-analysis by Sharp et al. (54) demonstrated pooled diagnostic yield of 84% for TBCB and 64% for forceps transbronchial biopsy. However, all pooled studies lacked a head-to-head comparison of these two procedures for the diagnosis of ILDs. They separately analyzed the studies on TBCB and forceps transbronchial biopsy and concluded that TBCB is associated with a diagnostic yield higher than forceps biopsy based on indirect comparison. Meta-analysis by Ganganah et al. (13) was the first study that pooled the studies directly comparing cryobiopsy and forceps biopsy for ILDs and lung tumors. They found that cryobiopsy group had a higher diagnostic yield than forceps group (91.6 vs. 73.13%). Moreover, significant differences were observed in the diagnostic yield between cryobiopsy group and forceps group in both ILDs and lung tumor subgroups. However, they have combined studies with endobronchial and peripheral lesions to analyze the efficacy and safety of cryobiopsy which may have introduced bias. The present meta-analysis addressed limitations of previous meta-analyses (13, 53–55). Sryma et al. (56) reported no significant difference in diagnostic yield between cryobiopsy and forceps biopsy, but in our study, we did find significant difference between the two modalities for the diagnosis of PPLs. In pooled analysis, they included one single-arm study (57) which may have affected the overall result.

The present study demonstrates that cryobiopsy yielded larger tissue specimens in comparison with forceps biopsy. This is consistent with the findings from recent studies and systematic reviews (13, 53–55). However, meta-analysis by Ganganah et al. (13) only pooled specimen area (mm^2^) using SMD and excluded the specimen size given in millimeter or centimeter. The SMD is used when measurements are on different scales, so that converting to SMD makes them scale-free. Our meta-analysis pooled all the studies with specimen size measured on different scales. Larger specimen with more alveolated tissue, architecturally well-preserved specimens, and, less crush artifact harvested by cryobiopsy compared with forceps biopsy may enable pathologists to provide a diagnosis more accurately. A previous meta-analysis (13) failed to pool the results of bleeding severity and only performed qualitative analysis. In the present analysis, we pooled the moderate to severe bleeding in the cryobiopsy group compared with the forceps biopsy group. It has been shown that compared with forceps biopsy, the rate of clinically relevant bleeding (moderate or severe) was higher after the cryobiopsy procedures. This finding is consistent with recent studies, which showed that cryobiopsy was associated with the high risk of bleeding compared with other modalities (12, 58, 59). The safety profile of cryobiopsy has perhaps been the largest concern limiting its widespread adoption. According to Maldonado et al. (52), the common complications of bronchoscopic cryobiopsy are bleeding, pneumothorax, and pneumomediastinum. Therefore, availability of ice cold saline, vasoconstrictive agent, electrocoagulation, and endobronchial blockers should be ensured in centers where pleural cryobiopsy is performed to control moderate to severe bleeding. Several definitions of bleeding have been used in published studies that compared cryobiopsy and forceps biopsy. Table 2 highlights the lack of uniformity in bleeding definitions among included studies in our systematic review and meta-analysis. There was substantial heterogeneity among included studies regarding the grading of bleeding severity, as there is lack of internationally accepted bleeding severity scale. Future studies should focus on head-to head comparison among different biopsy modalities, with a uniform, consistent definitions for quantifying bleeding risk.

Furthermore, the present study demonstrates that cryobiopsy was not associated with an increased risk of adverse events, such as pneumothorax compared with forceps biopsy (6.1 vs. 6.8%). Previous cryobiopsy meta-analyses (53–55) showed that the rate of pneumothorax varied between 6 and 10%. A recent study by Herth et al. (58) showed that TBCB had the pneumothorax rate of 6.6%, which is similar to standard forceps biopsy. A recent prospective cohort study (60) compared the effect of different cryoprobe types on the outcomes of TBCB found that the risk of pneumothorax can be influenced by procedure-related factors. However, probe size (2.4 vs. 1.9-mm) was not associated with significant differences in the risk of pneumothorax. Notably, significantly increased risk of pneumothorax was found with probe-to-pleura distances of <1 cm. Therefore, cryobiopsy procedure should be conducted routinely under fluoroscopy guidance with probe-to-pleura distances of >1 cm to decrease the risk of pneumothorax.

This meta-analysis has some limitations. First, the cryobiopsy procedures were performed by bronchopists depending on their experiences, the expertise of the operator and biopsy tools selection might have resulted in the high variability in diagnostic yield between individual studies. Second, there was variation in the definitions of bleeding severity, therefore we failed to pool the bleeding rate from all of the included studies. Third, our results are limited by the significant heterogeneity between studies, which could be caused by the diversity in cryobiopsy sampling protocols and inconsistent use of outcome definitions. Fourth, we were unable to perform a meta-regression analysis to test for variables associated with diagnostic yield and specimen size because of insufficient data.

## Conclusions

Despite the aforementioned limitations, the present systemic review and meta-analysis is the first, to our knowledge, the most comprehensive meta-analysis to date evaluating the efficacy and safety of cryobiopsy vs. forceps biopsy for the lung pathologies. The results of our study demonstrate that cryobiopsy is a safe and effective alternative to forceps biopsy. However, the quality of the available evidence is moderate, and well-designed RCTs comparing performance of cryobiopsy vs. forceps biopsy including consecutive patients and using valid reference standards is required to corroborate these findings and address knowledge gaps.

## Data Availability Statement

The original contributions presented in the study are included in the article/Supplementary Material, further inquiries can be directed to the corresponding author.

## Author Contributions

MG, GH, and SG: conceptualization. MG, GH, AP, and RZ: data curation. MG, AP, YL, and SG: formal analysis. MG and AP: investigation. MG, AP, and GH: project administration. SG and MG: supervision. SG, MG, RZ, and GH: validation and writing. MG, YL, RZ, and SG: visualization. MG, AP, and SG: review and editing. All authors contributed to the article and approved the submitted version.

## Funding

This study was supported by the Project of Chongqing Science and Technology Commission (cstc2017shmsA1143).

## Conflict of Interest

The authors declare that the research was conducted in the absence of any commercial or financial relationships that could be construed as a potential conflict of interest.

## Publisher's Note

All claims expressed in this article are solely those of the authors and do not necessarily represent those of their affiliated organizations, or those of the publisher, the editors and the reviewers. Any product that may be evaluated in this article, or claim that may be made by its manufacturer, is not guaranteed or endorsed by the publisher.
