# Supplementary material for: Efficacy and Safety of Cryobiopsy vs. Forceps Biopsy for Interstitial Lung Diseases, Lung Tumors, and Peripheral Pulmonary Lesions: An Updated Systematic Review and Meta-Analysis
Source: Front Med (Lausanne). 2022 Mar 10;9:840702. doi: 10.3389/fmed.2022.840702 (PMC8965605; doi:10.3389/fmed.2022.840702)
Supplement: Supplementary file 1 [file Data_Sheet_1.docx]

**Supplementary Material**

**Contents**

**Supplementary appendix 1.** Electronic search in PubMed database

**Supplementary Figure 1.** Risk of bias graph for RCTs

**Supplementary Figure 2.** Risk of bias summary for RCTs

**Supplementary Figure 3**. Funnel plots to detect publication bias for studies comparing diagnostic rate

**Supplementary Figure 4.** Forest plot of sensitivity analysis for overall diagnostic yield performed using the trim and fill method.

**Supplementary Figure 5.** Funnel plots to detect publication bias for studies comparing specimen size

**Supplementary Figure 6**. Forest plot of sensitivity analysis for specimen size performed using the trim and fill method

**Supplementary Figure 7.** Funnel plot of studies comparing cryobiopsy versus forceps biopsy for moderate to severe bleeding

**Supplementary Figure 8**. Forest plot comparing the incidence of pneumothorax between the cryobiopsy versus forceps biopsy group.

**Supplementary Figure 9.** Funnel plot to detect publication bias for incidence of pneumothorax

**Supplementary Table 1**. Study Quality Assessment for non-RCTs by Newcastle-Ottawa scale

**Supplementary Table 2**. Qualitative analysis of bleeding severity between the cryobiopsy versus forceps biopsy group.

**Supplementary Table 3**. Qualitative analysis of Specimen size obtained by cryobiopsy and forceps biopsy

**Supplementary appendix 1.** Electronic search in PubMed database

| ***Search ID*** | ***Search Terms*** |
| --- | --- |
| #1 | lung cancer[Title/Abstract] OR lung neoplasm[Title/Abstract] OR lung carcinoma[Title/Abstract] OR lung tumor[Title/Abstract] OR lung adenocarcinoma[Title/Abstract] |
| #2 | pulmonary nodule[Title/Abstract] OR peripheral pulmonary lesion[Title/Abstract] |
| #3 | interstitial lung diseases[Title/Abstract] OR diffuse parenchymal lung diseases[Title/Abstract] OR lung fibrosis[Title/Abstract] |
| #4 | ((lung cancer[Title/Abstract] OR lung neoplasm[Title/Abstract] OR lung carcinoma[Title/Abstract] OR lung tumor[Title/Abstract] OR lung adenocarcinoma[Title/Abstract]) OR (pulmonary nodule[Title/Abstract] OR peripheral pulmonary lesion[Title/Abstract])) OR (interstitial lung diseases[Title/Abstract] OR diffuse parenchymal lung diseases[Title/Abstract] OR lung fibrosis[Title/Abstract]) |
| #5 | Cryobiopsy[Title/Abstract] OR Cryoprobe biopsy[Title/Abstract] OR Cryotransbronchial biopsy[Title/Abstract] OR Transbronchial cryobiopsy[Title/Abstract] |
| #6 | #4 AND #5 |

# Supplementary Figure 1. Risk of bias graph for RCTs

**
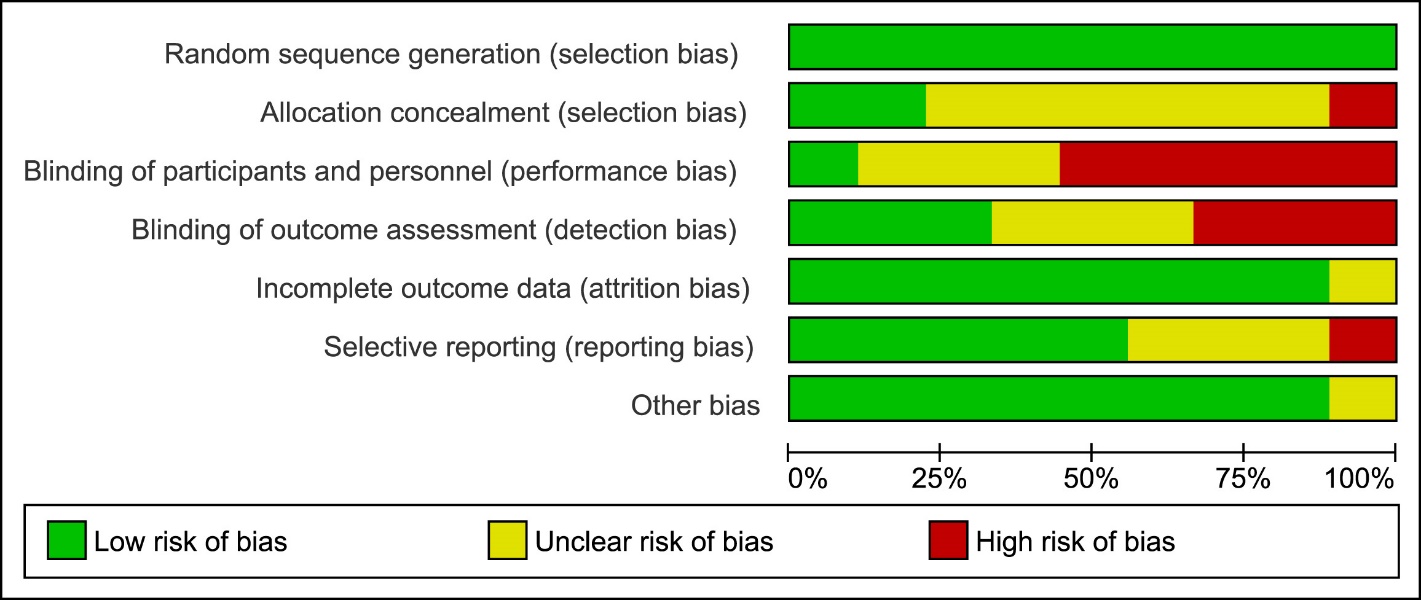
**

Authors' assessments of each risk of bias item are presented as percentages across all included randomized controlled trials.

**Supplementary Figure 2**. Risk of bias summary for included RCTs

**
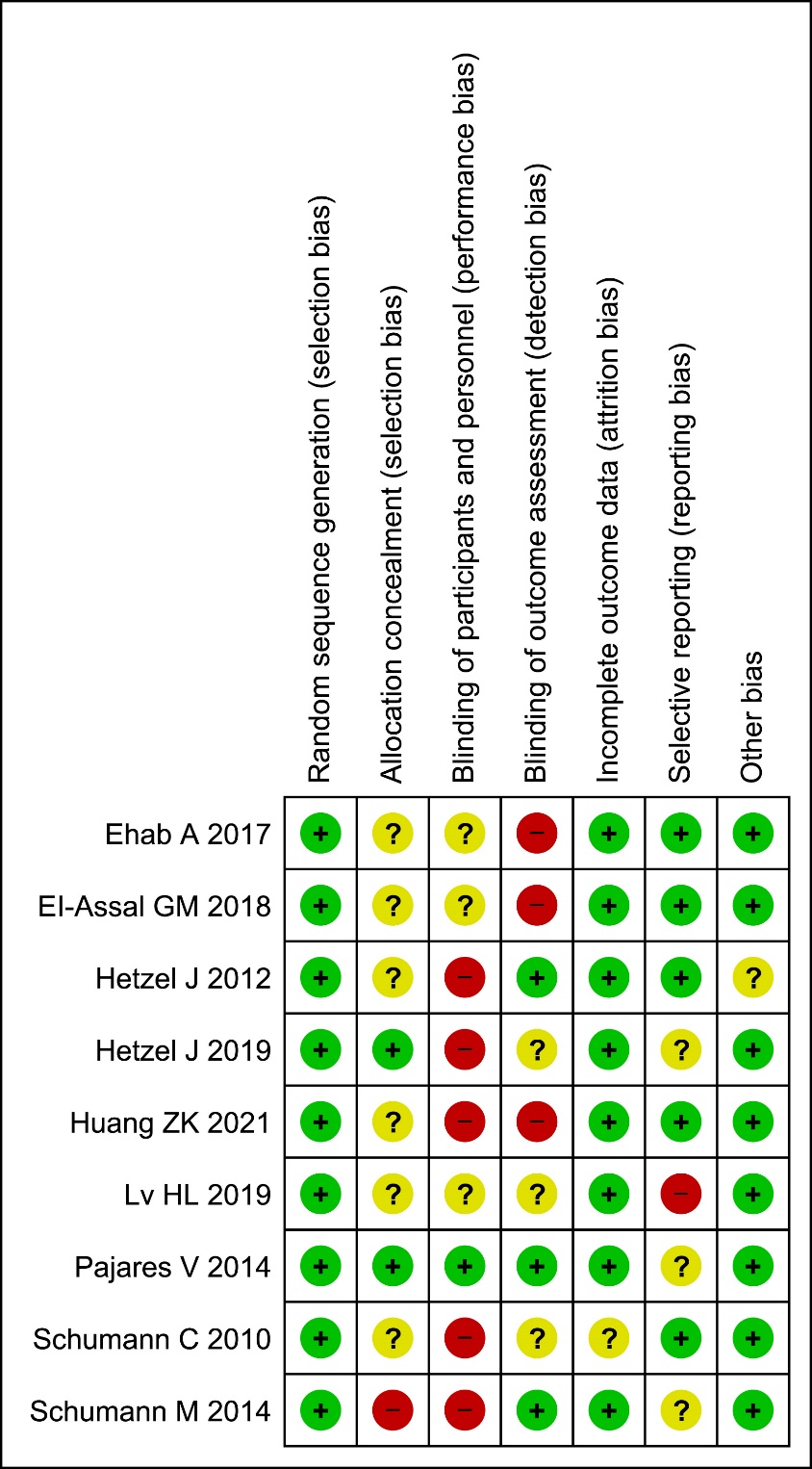
**

The judgments of the authors for each risk of bias item in the included randomized controlled trials. All studies had adequate random sequence generation. Blinding of participants and personnel (5 studies), blinding of outcome assessment (3 studies), allocation concealment (1 study), and selective reporting (1 study) were the main source of high risk of bias.

**Supplementary Figure 3.** Funnel plots to detect publication bias for studies comparing diagnostic rate (**A**) Funnel plot demonstrated asymmetry, (**B**) Trim and fill plot of publication bias. Ten studies (white circles) were included when the trim-and-fill method was applied.

**
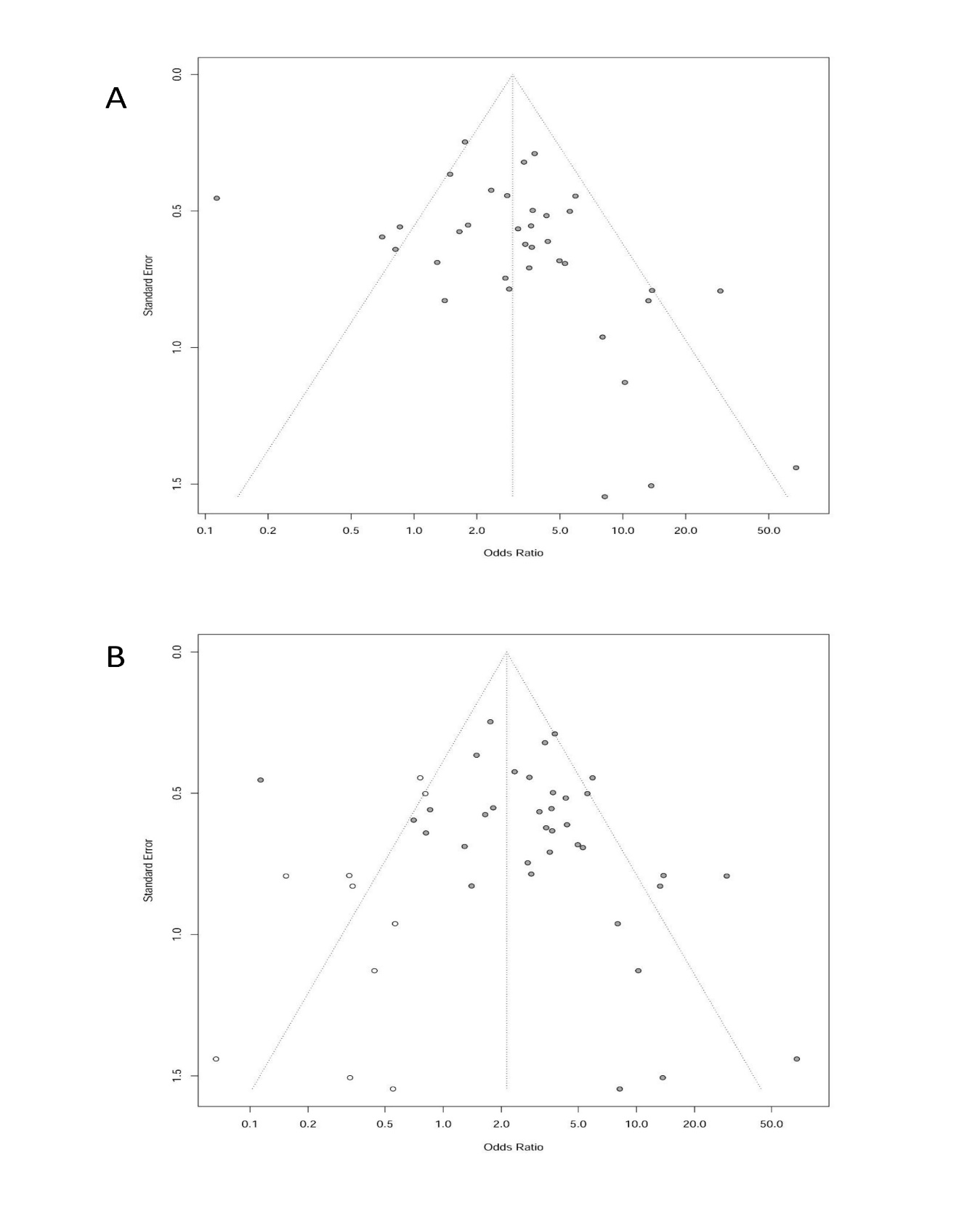
**

**Supplementary Figure 4.** Forest plot of sensitivity analysis for overall diagnostic yield performed using the trim and fill method

**
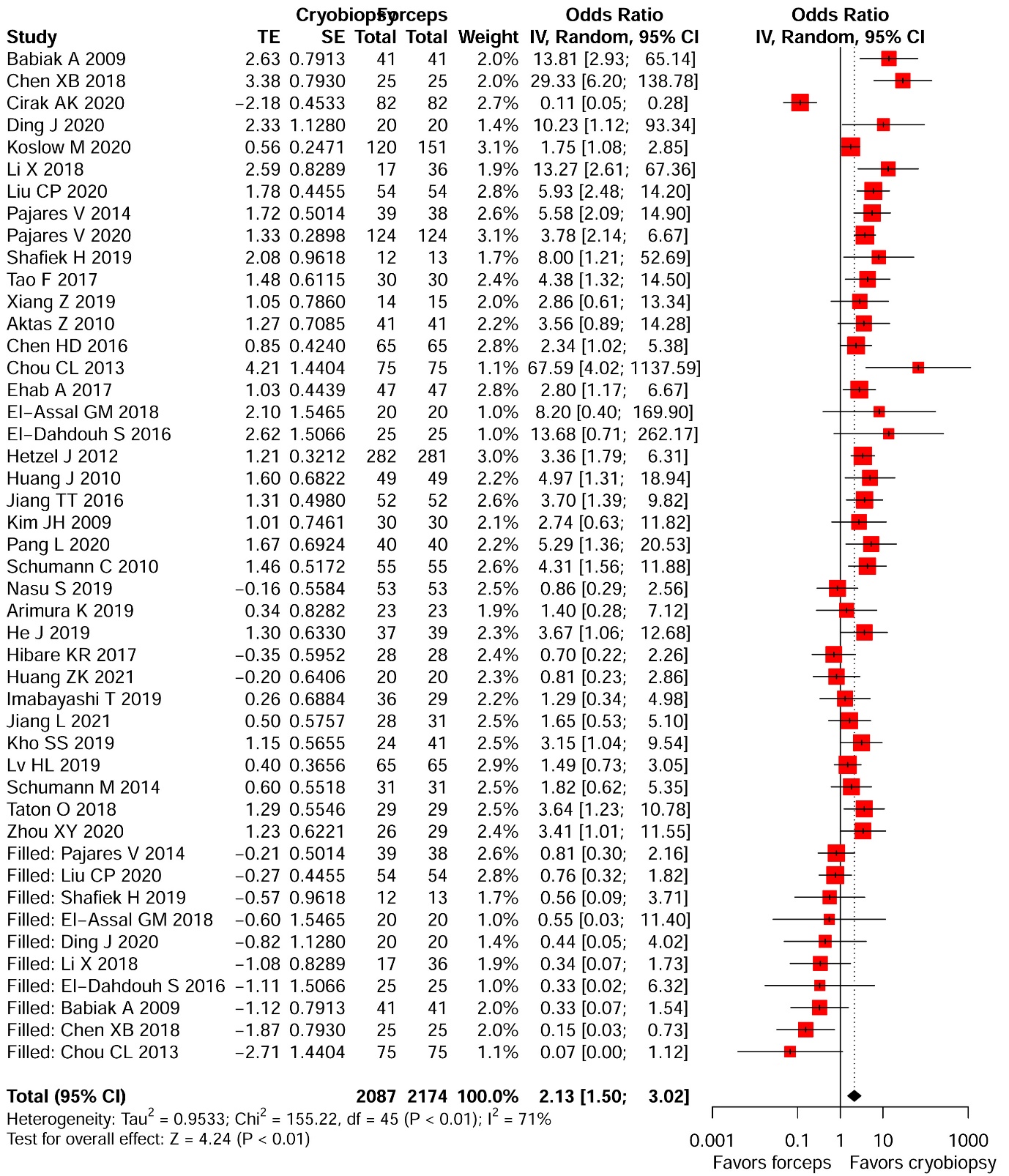
**

**Supplementary Figure 5.** Funnel plots to detect publication bias for studies comparing specimen size (A) Funnel plot showed asymmetry, (B) Trim and fill plot of publication bias resolved publication bias. Eight studies (white circles) were imputed when the trim-and-fill method was applied.

**
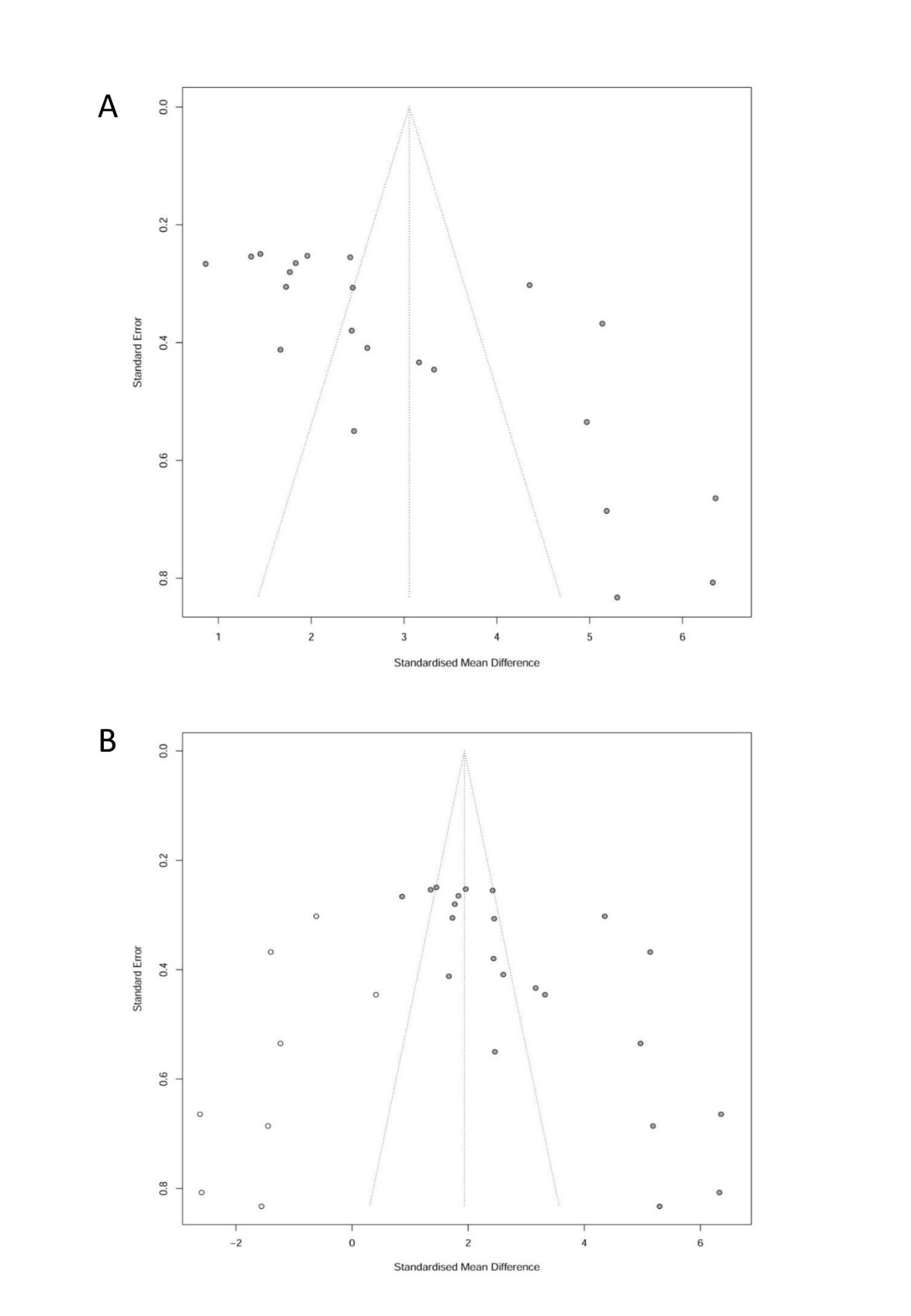
**

**Supplementary Figure 6.** Forest plot of sensitivity analysis for specimen size performed using the trim and fill method

**
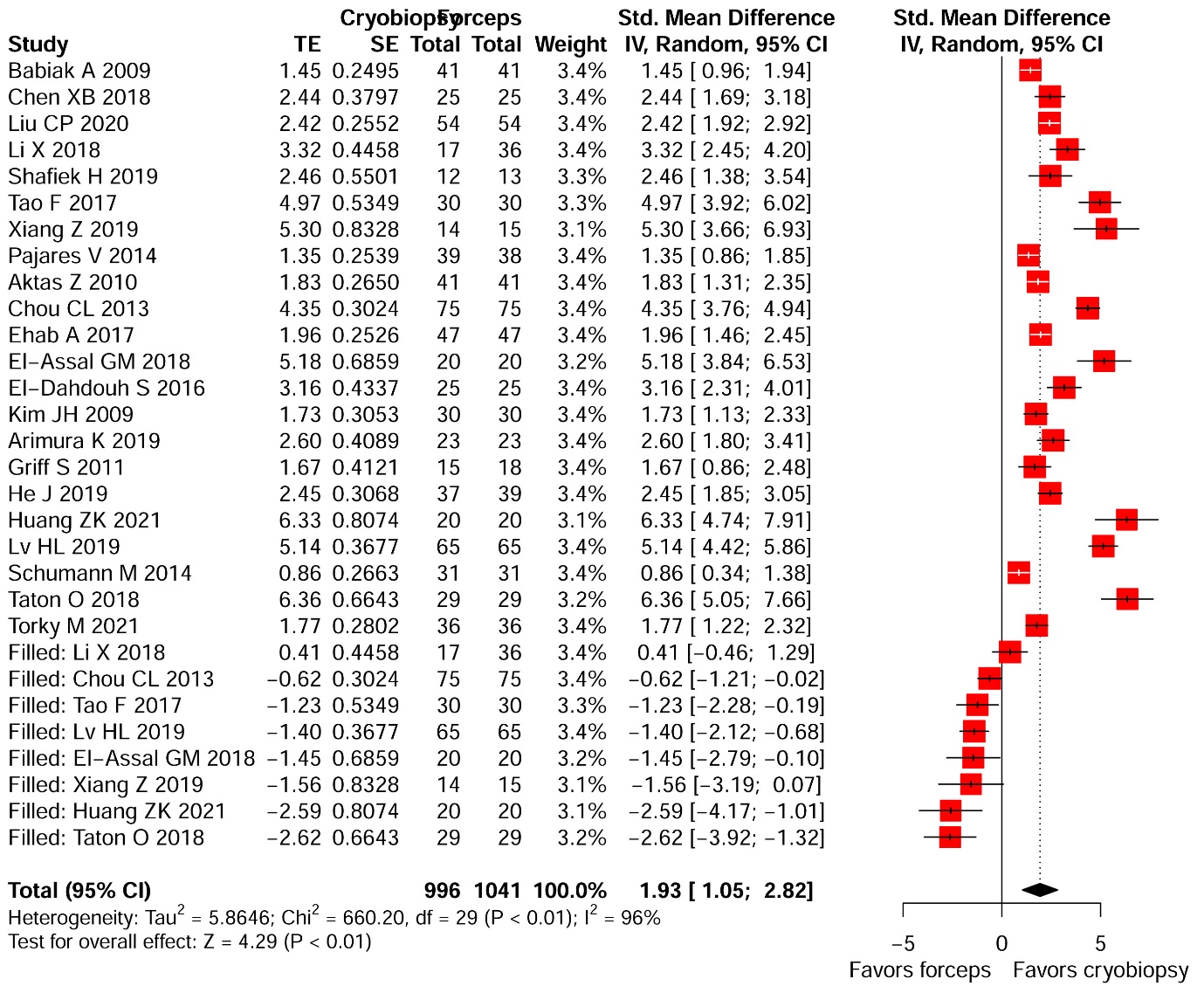
**

**Supplementary Figure 7.** Funnel plot of studies comparing cryobiopsy versus forceps biopsy for moderate to severe bleeding

**
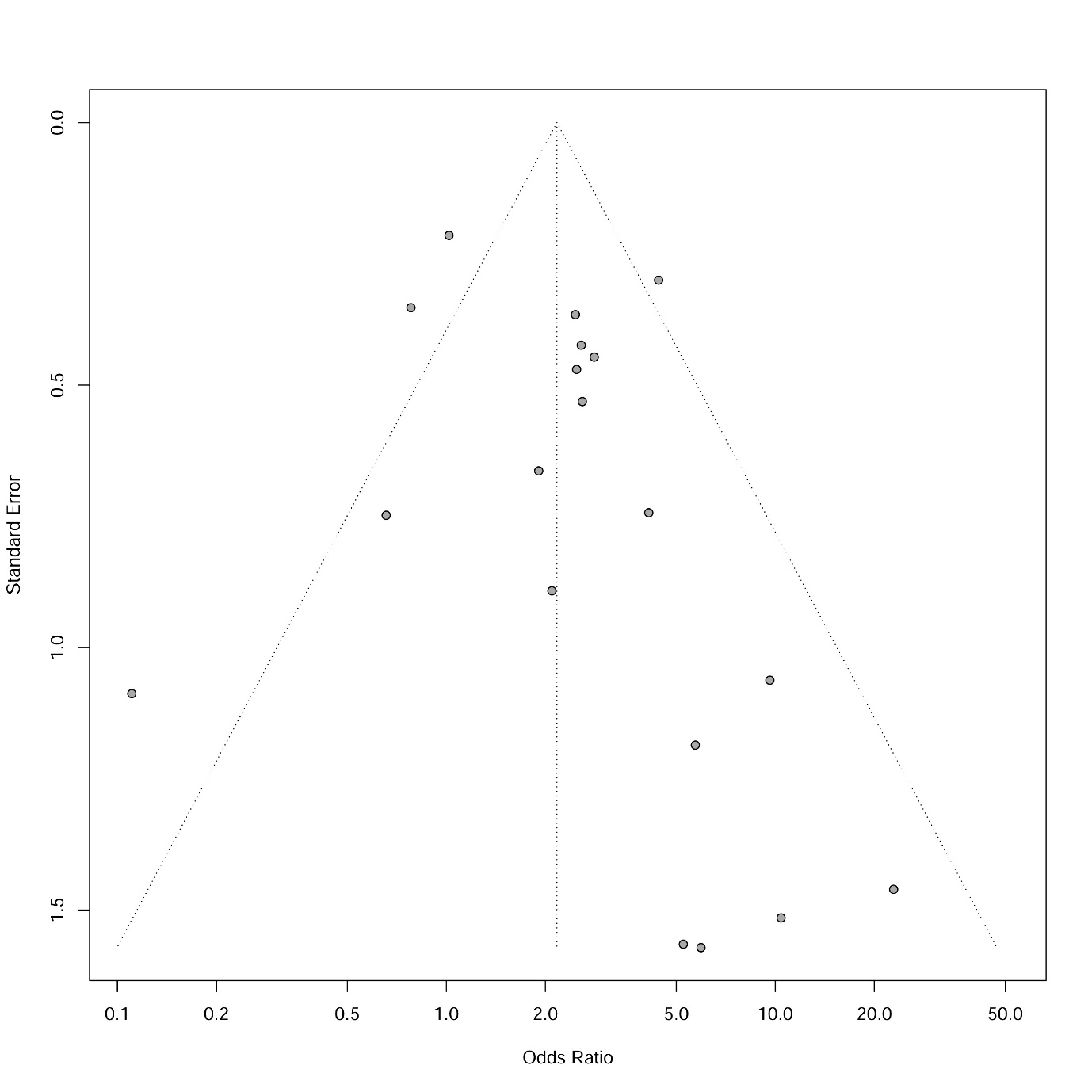
**

**Supplementary Figure 8**. Forest plot comparing the incidence of pneumothorax between the cryobiopsy versus forceps biopsy group.

**
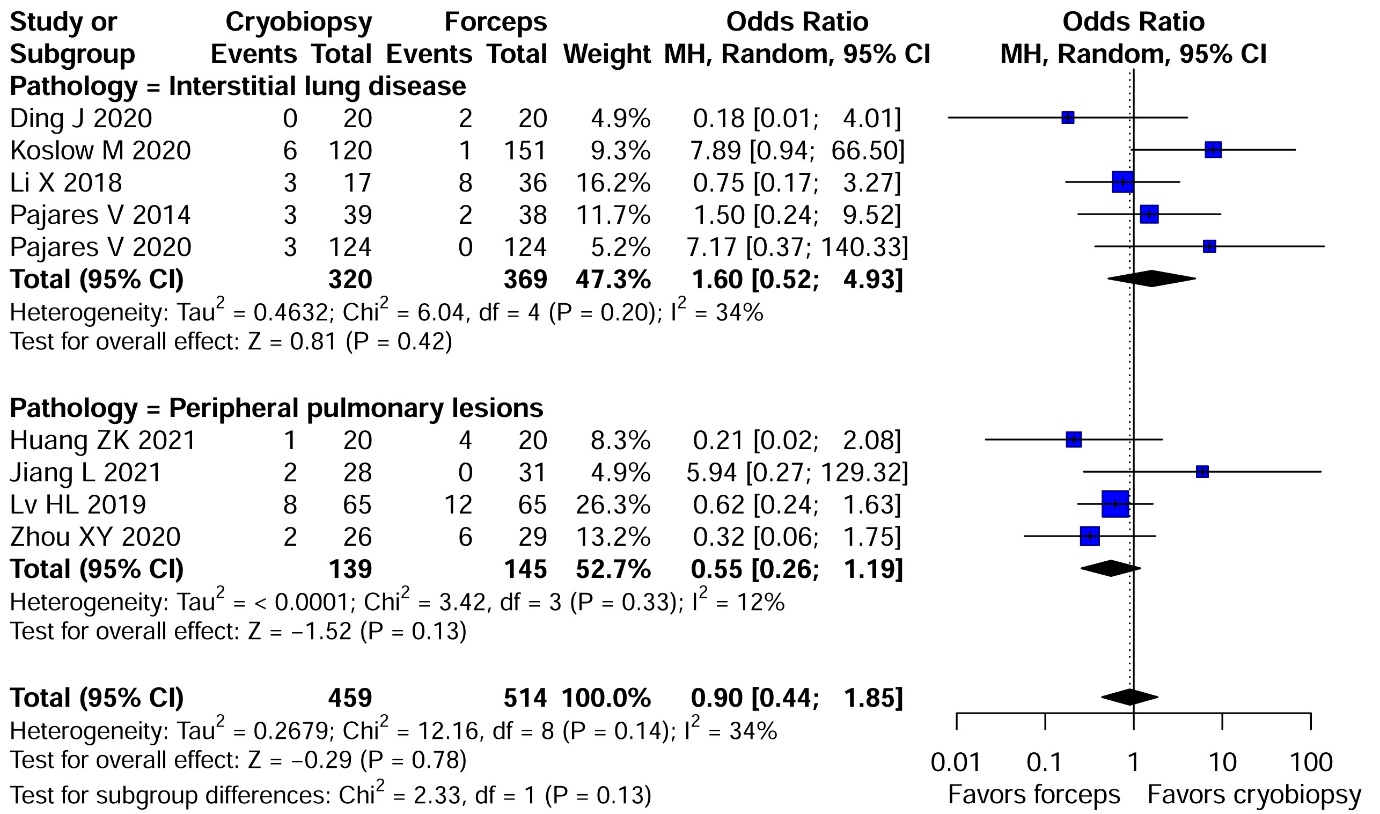
**

**Supplementary Figure 9**. Funnel plot to detect publication bias for incidence of pneumothorax


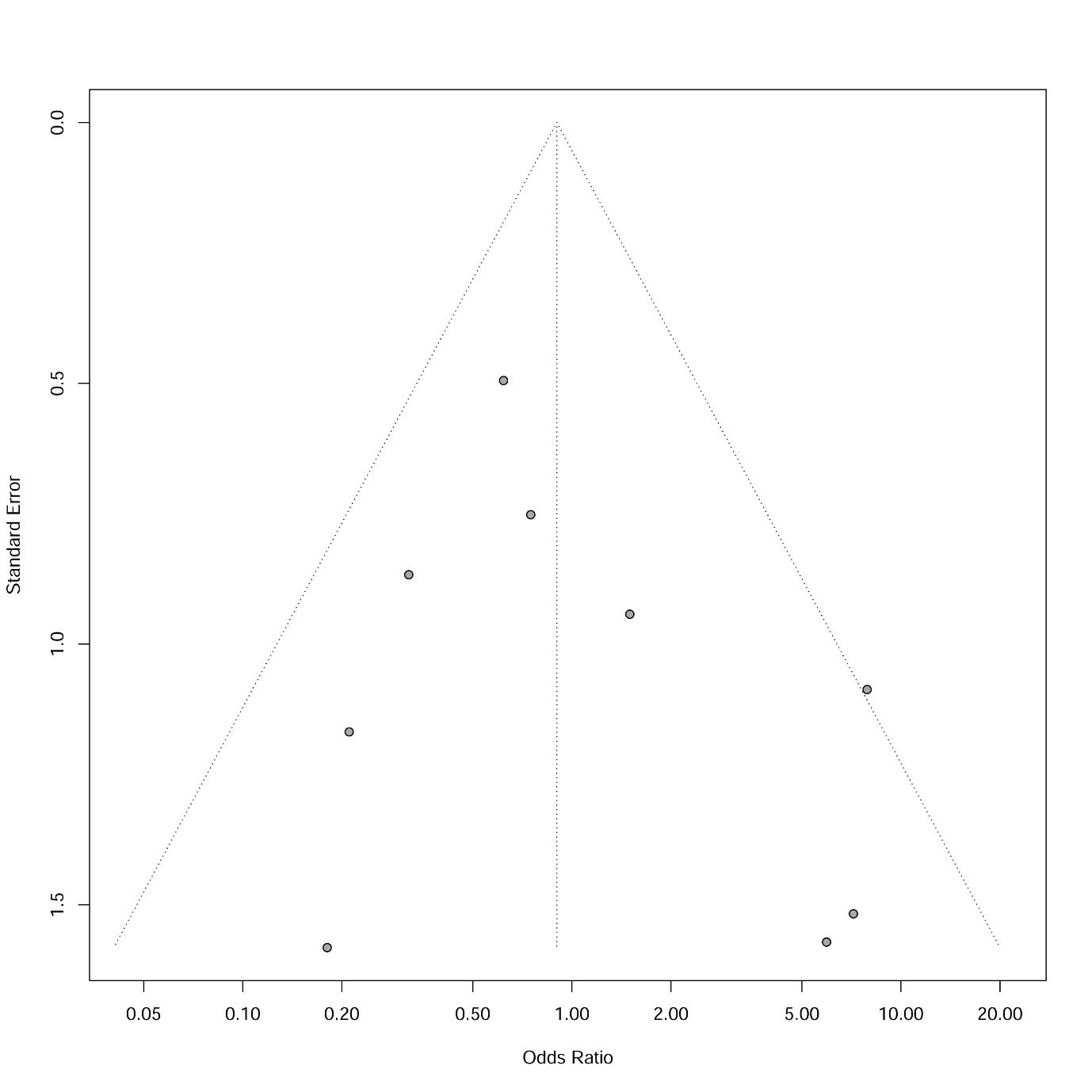


**Supplementary Table 1**. Study Quality Assessment for non-RCTs by Newcastle-Ottawa scale

| First author (year) | Selection Bias | Comparability | Exposure | Total Score | Quality |
| --- | --- | --- | --- | --- | --- |
| Aktas Z (2010) | 3 | 1 | 3 | 7 | High |
| Arimura K (2019) | 3 | 2 | 3 | 8 | High |
| Babiak A (2009) | 2 | 1 | 3 | 6 | Medium |
| Chen HD (2016) | 3 | 1 | 2 | 6 | Medium |
| Chen XB (2018) | 3 | 1 | 2 | 6 | Medium |
| Chou CL (2013) | 3 | 1 | 2 | 6 | Medium |
| Cirak AK (2020) | 3 | 1 | 2 | 6 | Medium |
| Ding J (2020) | 2 | 1 | 3 | 6 | Medium |
| El-Dahdouh S (2016) | 4 | 1 | 3 | 8 | High |
| Griff S (2011) | 3 | 1 | 2 | 6 | Medium |
| He J (2019) | 3 | 1 | 3 | 7 | High |
| Hibare KR (2017) | 3 | 1 | 2 | 6 | Medium |
| Huang J ( 2010) | 2 | 1 | 3 | 6 | Medium |
| Imabayashi T (2019) | 3 | 1 | 2 | 6 | Medium |
| Jiang L (2021) | 3 | 1 | 3 | 7 | High |
| Jiang TT (2016) | 2 | 1 | 3 | 6 | Medium |
| Kho SS (2019) | 3 | 2 | 2 | 7 | High |
| Kim HJ (2009) | 3 | 1 | 2 | 6 | Medium |
| Koslow M (2020) | 3 | 2 | 3 | 8 | High |
| Liu CP (2020) | 3 | 1 | 2 | 6 | Medium |
| Li X (2018) | 2 | 1 | 3 | 6 | Medium |
| Nasu S (2019) | 3 | 1 | 2 | 6 | Medium |
| Pajares V (2020) | 3 | 2 | 3 | 8 | High |
| Pang L (2020) | 3 | 1 | 3 | 7 | High |
| Shafiek H (2019) | 3 | 1 | 3 | 7 | High |
| Tao F (2017) | 2 | 2 | 3 | 7 | High |
| Taton O (2018) | 3 | 1 | 3 | 7 | High |
| Torky M (2021) | 2 | 1 | 3 | 6 | Medium |
| Xiang Z (2019) | 2 | 1 | 3 | 6 | Medium |
| Zhou XY (2020) | 3 | 1 | 2 | 6 | Medium |

NOS scores of 0–3, 4–6, and 7–9 indicates low, moderate, and high quality, respectively

**Supplementary Table 2**. Qualitative analysis of bleeding severity

| First author (year) | Definition of bleeding severity | Number of patients in CB, n (%) | Number of patients in FB, n (%) | *P* value |
| --- | --- | --- | --- | --- |
| Aktas Z (2010) | Mild : Ice-cold NaCl or adrenalin solution | 8 (19.5) | 9 (21.9) | >0.05 |
| CB:41, FB: 41 | Moderate: requiring APC | 2 (4.9) | 0 (0) |  |
|  | Severe: Hemodynamic instability | 0 (0) | 0 (0) |  |
| Chen HD (2016) | Mild : Blood loss <10mL | 31 (47.7) | 45 (7.7) |  |
| CB:65, FB:65 | Moderate: Blood loss 10- 50 ml | 30 (46.1) | 20 (30.7) | NR |
|  | Severe: Blood loss >50mL | 4 (6.1) | 0 (0) |  |
| Chen XB (2018) | Grade 0: No bleeding | 56 (52.8) | 86 (81.1) |  |
| CB:106, FB:106 | Grade 1: negative pressure suction can stop bleeding by itself without other hemostatic measures | 41 (38.7) | 17 (16) | <0.05 |
|  | Grade 2: Need to locally inject frozen saline, epinephrine or balloon to stop bleeding | 9 (8.5) | 3 (2.8) |  |
|  | Grade 3: severe bleeding causing abnormal hemodynamics and respiratory function | 0 (0) | 0 (0) |  |
| Ehab A (2017) | No bleeding | 28 (59.6) | 35 (74.5) |  |
| CB:47, FB:47 | Mild : Ice-cold NaCl or adrenalin solution | 15 (31.9) | 10 (21.3) | 0.063 |
|  | Moderate: requiring APC | 4 (8.5) | 2 (4.3) | 0.5 |
|  | Severe: Hemodynamic instability, ICU admission | 0 (0) | 0 (0) |  |
| El-Assal GM (2018) | Mild | 11 (55) | 14 (70) | 0.42 |
| CB:20, FB:20 | Moderate | 8 (40) | 5 (25) |  |
|  | Massive | 1 (5) | 1 (5) |  |
| He J (2019) | Mild to moderate | 15 (40.5) | 7 (17.9) |  |
| CB: 37, FB: 39 | Severe | 0 (0) | 0 (0) |  |
| Hetzel J (2012) | No bleeding | 59 (19.9) | 91 (30.6) | 0.009 |
| CB: 296, FB: 297 | Mild: Controlled by suctioning | 183 (61.8) | 153 (51.5) |  |
|  | Severe: need instillation of ice-cold saline or a diluted vasoconstrictive drug, balloon tamponade, APC etc. | 54 (18.2) | 53 (17.8) |  |
| Hetzel J (2019) | None | 98 (27.3) | 186 (51.8) |  |
| CB: 359, FB: 359 | Mild : self-limiting bleeding, manageable with suction alone and without the need for any specific intervention | 203 (56.5) | 158 (44) | < 0.001 |
|  | Moderate: use of additional intervention such as instillation of ice-cold saline or vasoconstrictive drugs, or transient balloon tamponade | 54 (15) | 15 (4.2) | < 0.001 |
|  | Severe: Prolonged monitoring or intensive care therapy after the procedure was necessary or fatal bleeding | 4 (1.1) | 0 (0) |  |
| Huang J (2010) | Mild : Blood loss <10mL | 11 (22.4) | 22 (44.9) | NR |
| CB: 49, FB: 49 | Moderate: Blood loss 10- 50 ml | 36 (73.5) | 27 (55.1) |  |
|  | Severe: Blood loss >50mL | 2 (4) | 0 (0) |  |
| Huang ZK (2021) | Mild | 0 (0) | 1 (5) | <0.05 |
| CB: 20, FB: 20 | Moderate | 0 (0) | 0 (0) |  |
|  | Severe | 0 (0) | 0 (0) |  |
| Jiang L (2021) | Light | 4 (14.28) | 2 (6.45) | 0.32 |
| CB: 28, FB: 31 | Severe: Required intravenous hemostatic drug administration, cessation of procedure, and inflation of the bronchial blocking balloon | 2 (7.14) | 0 (0) | 0.13 |
|  | Fatal: Associated with cardiopulmonary instability, transfusion of packed RBCs, or surgical intervention | 0 (0) | 0 (0) |  |
| Jiang TT (2016) | Mild | 13 (25) | 24 (46.1) |  |
| CB: 52, FB: 52 | Moderate | 38 (73.1) | 28 (53.8) |  |
|  | Severe | 1 (1.9) | 0 (0) |  |
| Kho SS (2019) | Mild: Controlled by suctioning with or without local instillation of adrenaline | 15 (39.5) | 5 (6.6) | <0.001 |
| CB: 38, FB: 76 | Moderate: Involved prolonged balloon blockade | 3 (7.9) | 1 (1.3) |  |
|  | Severe: Rrequired additional intervention, such as bronchial artery embolisation, transfusion, intubation or emergency surgical treatment | 0 (0) | 0 (0) |  |
| Koslow M (2020)  CB: 120, FB: 151 | Significant bleeding: Prolonged or repeated balloon occlusion, use of adjunct measures (eg, cold saline), or blood product transfusion | 8 (7) | 0 (0) | 0.001 |
| Li X (2018) | Grade 0: No bleeding | 3 (17.6) | 4 (9) |  |
| CB: 17, FB: 36 | Grade 1: negative pressure suction can stop bleeding by itself without other hemostatic measures | 13 (76.5) | 19 (52.8) | 0.02 |
|  | Grade 2: Need to locally inject frozen saline, epinephrine or balloon to stop bleeding | 1 (5.9) | 13 (36.1) |  |
|  | Grade 3: severe bleeding causing abnormal hemodynamics and respiratory function, Endoscopic treatment or surgical intervention is required | 0 (0) | 0 (0) |  |
| Lv HL (2019) | Mild: Bleeding stops itself | 15 (23.07) | 22 (32.84) |  |
| CB: 65, FB: 67 | Moderate: Need to locally inject frozen saline, epinephrine | 28 (43.08) | 32 (47.76) | >0.05 |
|  | Severe: Prolonged monitoring or intensive care therapy after the procedure was necessary or fatal bleeding | 0 (0) | 0 (0) |  |
| Pajares V (2014) | Grade 0: No bleeding | 5 (12.8) | 8 (21.1) | 0.068 |
| CB: 39, FB: 38 | Grade 1: Bleeding requiring suction to clear but no other endoscopic procedures | 12 (30.8) | 17 (44.7) |  |
|  | Grade 2 : Bleeding requiring endoscopic procedures (bronchial occlusion-collapse and/or instillation of ice-cold saline) | 22 (56.4) | 13 (34.2) |  |
|  | Grade 3: Severe bleeding not controlled endoscopically, causing haemodynamic or respiratory instability, requiring surgical interventions or admission to ICU) | 0 (0) | 0 (0) |  |
| Pajares V (2020) | Grade 0 : No bleeding | 44 (35.5) | 70 (56.4) | 0.0001 |
| CB: 124, FB: 124 | Grade 1: Mild bleeding, i.e., some observed blood remains but endoscopic intervention not necessary); | 51 (42.1) | 36 (29.8) |  |
|  | Grade 2: Slight bleeding, i.e., bleeding requires balloon occlusion and stops in <3 minutes; | 21 (17.4) | 17 (14) |  |
|  | Grade 3: Moderate bleeding, i.e., bleeding requires balloon occlusion, needs >3 minutes to bring under control and requires suspension of the procedure | 8 (6.5) | 1 (0.8) |  |
|  | Grade 4: Severe bleeding, i.e., bleeding is endoscopically uncontrollable, causes haemodynamic or respiratory instability and requires suspension of the procedure | 1 (0.8) | 0 (0) |  |
| Shafiek H (2019)  CB: 12, FB: 13 | Mild bleeding | 1 (8.3) | 0 (0) |  |
| Tao F (2017) | Mild: Bleeding stops itself | 11 (36.67) | 18 (60) |  |
| CB: 30, FB: 30 | Moderate: Need to locally inject frozen saline, epinephrine | 19 (63.33) | 12 (40) | >0.05 |
|  | Severe: Prolonged monitoring or intensive care therapy after the procedure was necessary | 0 (0) | 0 |  |
| Taton O (2018) | Grade 0: no bleeding occurred; | 14 (48.3) | 27 (93.1) |  |
| CB: 29, FB: 29 | Grade 1: Bleeding stopped within five minutes either spontaneously or by inflation of the Fogarty balloon | 11 (38) | 2 (7) | 0.005 |
|  | Grade 2 : Bleeding was prolonged for more than five minutes or needed cold saline instillation | 4 (14) | 0 (0) | 0.043 |
|  | Grade 3 : Bleeding required embolization, selective bronchial intubation, transfusion, or admission in the ICU | 0 (0) | 0 (0) |  |
| Xiang Z (2019) | Mild: Bleeding stops itself | 8 (57.14) | 7 (46.67) | 0.138 |
| CB: 14, FB: 15 | Moderate: Need to locally inject frozen saline, epinephrine | 6 (42.86) | 8 (53.33) | 0.138 |
|  | Severe: Prolonged monitoring or intensive care therapy after the procedure was necessary | 0 (0) | 0 (0) |  |
| Zhou XY (2020) | Minor: Blood loss ≤5 mL | 24 (92.3) | 27 (93.1) |  |
| CB: 26, FB: 29 | Mild : Blood loss > 5 ≤20 m | 2 (7.7) | 2 (6.9) | NR |
|  | Moderate: Blood loss > 20 ≤100 m | 0 (0) | 0 (0) |  |
|  | Severe: Blood loss > 100 mL | 0 (0) | 0 (0) |  |

CB, cryobiopsy; FB, forceps biopsy; APC, argon plasma coagulation; ICU, intensive care unit, NR: not reported

**Supplementary Table 3.** Qualitative analysis of Specimen size obtained by cryobiopsy and forceps biopsy

| **First author (year)** | **Specimen size cryobiopsy** | **Specimen size forceps biopsy** |
| --- | --- | --- |
| Aktas Z (2010) | 0.8 (0.3-4) cm (median size, range) | 0.2 (0.1 -1) cm (median size, range) |
| Arimura K (2019) | 0.078 ± 0.008 cm^3^ (Volume ± SEM) | 0.003 ± 0.0003 cm^3^ (Volume ± SEM) |
| Babiak A (2009) | 15.11 (2.15-54.15) mm^2^ (median area, range) | 5.82 (0.58-20.88) mm^2^ (median area, range) |
| Chen HD (2016) | 12.35 mm^2^ (mean area) | 5.68 mm^2^ (mean area) |
| Chen XB (2018) | 12.3 ± 4.9 mm^2^ (mean area ± SD) | 3.1 ± 1.9 mm^2^ (mean area ± SD) |
| Chou CL (2013) | 13.8 ± 3.8 mm (mean size ± SD) | 1.9 ± 0.6 mm (mean size ± SD) |
| Ehab A (2017) | 5.9 ± 2.3 mm (mean size ± SD) | 2.5 ± 0.8 mm (mean size ± SD) |
| El-Assal GM (2018) | 0.36 ± 0.09 cm (mean diameter ± SD) | 1.18 ± 0.20 cm (mean diameter ± SD) |
| El-Dahdouh S (2016) | 1.4 ± 0.29 cm (mean size ± SD) | 0.5 ± 0.27 cm (mean size ± SD) |
| Griff S (2011) | 17.1 ± 10.7 mm^2^ (mean area ± SD) | 3.8 ± 4 mm^2^  (mean area ± SD) |
| He J (2019) | 10.5 ± 4.2 mm^2^ (mean area ± SD) | 2.9 ± 1.3 mm^2^ (mean area ± SD) |
| Huang ZK (2021) | 3.94 ± 0.35 mm (mean diameter ± SD) | 1.95 ± 0.26 mm (mean diameter ± SD) |
| Kim HJ (2009) | 6 ± 3 mm (mean size ± SD) | 2 ± 1.2 mm (mean size ± SD) |
| Liu CP (2020) | 3.3 ± 1.3 mm^2^ (mean area ± SD) | 1.0 ± 0.3 mm^2^ (mean area ± SD) |
| Li X (2018) | 44.2 ± 17.2 mm^2^ (mean area ± SD) | 7.5 ± 6.1 mm^2^ (mean area ± SD) |
| Lv HL (2019) | 4.02 ± 0.48 mm (mean diameter ± SD) | 1.69 ± 0.42 mm (mean diameter ± SD) |
| Pajares V (2014) | 4.1 ± 1.5 mm (mean diameter ± SD)  14.7 ± 11 mm^2^ (mean area ± SD) | 1.8 ± 1 mm (mean diameter ± SD)  3.3 ± 4.1 mm^2^ (mean area ± SD) |
| Pang L (2020) | 8.7 mm (mean size) | 3.7 (mean size) |
| Schumann C (2010) | 10.4 mm^2^ (mean area) | 5.2 mm^2^ (mean area) |
| Schumann M (2014) | 11.17 mm^2^ (1.25– 38.59) mean area (range) | 4.69 mm^2^ (0.53–22) mean area (range) |
| Shafiek H (2019) | 3.88 ±1.19 mm (mean size ± SD) | 1.46 ± 0.66 mm (mean size ± SD) |
| Tao F (2017) | 3.91 ± 0.43 mm (mean diameter ± SD) | 1.72 ± 0.44 mm (mean diameter ± SD) |
| Taton O (2018) | 5.3 ± 0.7 mm (mean diameter ± SD) | 1.1 ± 0.6 mm (mean diameter ± SD) |
| Torky M (2021) | 7.3 ± 2.1 mm (mean diameter ± SD)  38.6 ± 20.4 mm^2^ (mean area ± SD) | 3.9 ± 1.6 mm (mean diameter ± SD)  12.7 ± 1.7 mm^2^ (mean area ± SD) |
| Xiang Z (2019) | 3.89±0.48 mm (mean diameter ± SD) | 1.88±0.22 mm (mean diameter ± SD) |
